# Intercropping improves faba bean photosynthesis and reduces disease caused by *Fusarium commune* and cinnamic acid-induced stress

**DOI:** 10.1186/s12870-024-05326-8

**Published:** 2024-07-09

**Authors:** Wenhao Yang, Zhenyu Zhang, Tingting Yuan, Yu Li, Qian Zhao, Yan Dong

**Affiliations:** College of Resources and Environment, Yunnan Agricultural University, No. 452 Fengyuan, Kunming, Yunnan, 650500 China

**Keywords:** Soil-borne disease, Autotoxicity, Plant growth, Cultural control

## Abstract

Modern intensive cropping systems often contribute to the accumulation of phenolic acids in the soil, which promotes the development of soilborne diseases. This can be suppressed by intercropping. This study analyzed the effects of intercropping on Fusarium wilt based on its effect on photosynthesis under stress by the combination of *Fusarium commune* and cinnamic acid. The control was not inoculated with *F. commune*, while the faba bean plants (*Vicia faba* L.) were inoculated with this pathogen in the other treatments. The infected plants were also treated with cinnamic acid. This study examined the development of Fusarium wilt together with its effects on the leaves, absorption of nutrients, chlorophyll fluorescence parameters, contents of photosynthetic pigments, activities of photosynthetic enzymes, gas exchange parameters, and the photosynthetic assimilates of faba bean from monocropping and intercropping systems. Under monocropping conditions, the leaves of the plants inoculated with *F. commune* grew significantly less, and there was enhanced occurrence of the Fusarium wilt compared with the control. Compared with the plants solely inoculated with *F. commune*, the exogenous addition of cinnamic acid to the infected plants significantly further reduced the growth of faba bean leaves and increased the occurrence of Fusarium wilt. A comparison of the combination of *F. commune* and cinnamic acid in intercropped wheat and faba bean compared with monocropping showed that intercropping improved the absorption of nutrients, increased photosynthetic pigments and its contents, electron transport, photosynthetic enzymes, and photosynthetic assimilates. The combination of these factors reduced the occurrence of Fusarium wilt in faba bean and increased the growth of its leaves. These results showed that intercropping improved the photosynthesis, which promoted the growth of faba bean, thus, reducing the development of Fusarium wilt following the stress of infection by *F. commune* and cinnamic acid. This research should provide more information to enhance sustainable agriculture.

**Supplementary Information:**

The online version contains supplementary material available at 10.1186/s12870-024-05326-8.

## Introduction

The continuous growth of crops on the same land has become widespread owing to reductions in the availability and area of arable land [1–3]. However, growing the same crop for many years can result in continuous cropping obstacles, also known as yield decline. The condition manifests as weaker plant growth, lower yields, decreased quality and more soilborne diseases [4, 5]. There are many reasons for this phenomenon, including diseases and pests, an imbalance in the soil physicochemical properties, and the accumulation of allelopathic compounds [2, 6–10]. Among them, the synergistic effect of various factors on yield decline has been a topic of wide concern [2, 4]. Of particular concern is the synergistic effect between soilborne pathogens and allelopathic autotoxicity. Some studies have shown that exogenous application of cinnamic acid to cucumber (*Cucumis sativus*) plants that had been inoculated with *F. oxysporum* f. sp. *cucumerinum*, the causal agent of cucumber Fusarium wilt, resulted in a decrease in photosynthesis and leaf area in cucumber and promoted the occurrence of this disease [11]. It is apparent that the synergistic effect of soilborne pathogens and allelopathic autotoxicity can aggravate the occurrence of these diseases.

Currently, soilborne diseases are generally controlled by grafting, chemical, and physical treatments in agricultural production. Some studies have reported that grafting pumpkin (*Cucurbita pepo*) and gourd (*Cucurbita* spp.) rootstocks on watermelon (*Citrullis lanatus*) seedlings reduced the incidence of Fusarium wilt of watermelon caused by *F. oxysporum* f. sp. *niveum* [12–14], but it has not been widely used by growers owing to the high levels of skill and the cost required for grafting [15]. Treating the soil with steam to disinfect it at high temperatures can control soilborne diseases [16]. However, this technique can easily lead to secondary colonization and a large accumulation of soilborne pathogens, which can have grave effects on the growth of subsequent crops [17]. A concentration of 98% dazomet was used to disinfect the soil, which substantially decreased the amount of *Fusarium* propagules [18]. However, its use to eliminate pathogens also kills beneficial microorganisms, disrupts the ecological balance of microbes in the soil, and aggravates environmental pollution. There are limitations in all of these control methods; thus, the development of management strategies using benign environmental techniques to control soilborne disease is important [2, 19, 20].

The simultaneous planting of two or more crops is defined as intercropping, and it serves as a green and efficient pattern of cultivation that suppresses soilborne diseases [19, 21]. Intercropping can effectively alleviate soilborne diseases and promote the growth of crops. This has been observed in the intercropping of soybean (*Glycine max*) and corn (*Zea mays*) [21], cucumber and wheat (*Triticum aestivum*) [22], and tomato (*Solanum lycopersicum*) and onion (*Allium cepa*) [19]. Currently, the mechanism of the alleviation of soilborne diseases by intercropping has primarily been studied in different systems, including the growth of soilborne pathogenic fungi, rhizosphere microflora, community structure and recruitment of beneficial microorganisms. For example, intercropping with rice (*Oryza sativa*) and watermelon increased the population of bacteria and decreased the populations of *Fusarium* and other fungi in the rhizosphere compared with the monocropping of watermelon [23]. Similarly, the intercropping of wheat and cucumber improved the diversity of bacterial community, increased the abundance of beneficial species of *Pseudomonas*, and decreased the development of Fusarium wilt in cucumber compared with plants that had been grown in a monoculture [22]. The efficiency of use of natural resources is also increased by intercropping [24, 25]. Photosynthesis is the basic physicochemical process that enables plants to survive and grow [26–28]. This process is heavily influenced by biotic and abiotic factors, and almost all the damage from stress can be attributed to significant influences on photosynthesis [29, 30]. However, few studies have been conducted on how intercropping regulates photosynthesis of the host to alleviate soilborne diseases, particularly under interaction between soilborne pathogenic fungi and autotoxic compounds.

Faba bean (*Vicia faba* L.) is widely cultivated and provides a large amount of protein, which benefits health worldwide [31]. However, it is highly susceptible to infection by *Fusarium*, which results in an increase in the prevalence of soilborne wilt during the process of continuously planting faba bean [4, 32]. Wheat is frequently cultivated with faba bean to alleviate the wilt on this crop in southwest China, including Yunnan Province [33]. There is little information on the influence of intercropping on Fusarium wilt of faba bean, particularly in terms of the synergistic actions between *Fusarium* and autotoxic compounds. Cinnamic acid has been shown to be the principal autotoxic compound produced by the roots of faba bean and can remain stable in the soil [2, 34]. This study hypothesized that the synergy between *F. commune* and cinnamic acid promotes Fusarium wilt, and this can be mitigated by intercropping faba bean with wheat. This process can be studied by analyzing alterations in photosynthesis. Therefore, the primary goals of this research were as follows: (1) to evaluate the influence of *F. commune* and cinnamic acid on the development of Fusarium wilt and whether these could be mitigated by intercropping with wheat; (2) to evaluate the effects of *F. commune* and cinnamic acid on the absorption of nutrients by the host and how these were influenced by intercropping; (3) to evaluate the effects of *F. commune* and cinnamic acid on the photosynthetic pigments, electron transport and photosynthetic enzymes of the host and how these were influenced by intercropping; and (4) to evaluate the effects of *F. commune* and cinnamic acid on the photosynthetic assimilates of hosts and how these were influenced by intercropping. This research should provide more information to enhance sustainable agriculture.

## Materials and methods

### Test materials

This research included faba bean (*Vicia faba* L.) variety “89–147”and wheat (*Triticum aestivum* L.) variety “Yunmai 53” were provided by the Yunnan Academy of Agricultural Sciences (Kunming, China). The pathogenic fungus *Fusarium commune* that infects faba bean plants was used in the study [35]. The spores were collected from the culture plate, which was filtered through four layers of gauze to make a suspension of 1 × 10^6^ CFU·mL^-1^, which was used to inoculate the plants [36].

### Experimental design

A hydroponic experiment was conducted in the greenhouse at Yunnan Agricultural University (Kunming, China) from September to December 2021. The field trials were conducted between October 2020 and May 2021 in Efeng Village, Eshan County, Yuxi City, Yunnan Province, China (24° 11′N, 102° 24′E; above sea level 1,540 m). The faba bean rhizosphere soil at the tested field was collected 7 years after continuous cultivation as described by Yang et al. [2]. The contents of cinnamic acid and its derivatives, including ferulic acid and vanillic acid, had previously been shown to be 48.26 µg∙g^− 1^ (48.26 mg∙kg^− 1^) in the faba bean rhizosphere at harvest [2]. These experiments were based on the ratio of water: general soil density (1:2.65). In addition, previous studies have demonstrated that phenolic acids can be metabolized by soil microorganisms, which leads to their degradation [37]. We selected a hydroponic system to simulate the role of the synergy between *Fusarium commune* and cinnamic acid. This study was conducted in a hydroponic system that utilized a randomized block design that examined multiple factors with the following treatments: (1) faba bean monocropping (M) with no inoculation of *F. commune*: Ct; (2) faba bean monocropping (M) inoculated with *F. commune* without the addition of cinnamic acid: Fc; (3) faba bean monocropping (M) inoculated with *F. commune* and treated with 50 mg·L^− 1^ of cinnamic acid: Fc + C1; (4) faba bean monocropping (M) inoculated with *F. commune* and treated with 100 mg·L^− 1^ of cinnamic acid: Fc + C2; (5) faba bean monocropping (M) inoculated with *F. commune* and treated with 200 mg·L^− 1^ of cinnamic acid: Fc + C3; and (6–10) were treated as described above but were subjected to intercropping with faba bean and wheat (I). Thus, there were 10 treatments combinations and each treatment including five plastic basins (25 cm upper diameter, 16 cm height and 13 cm lower diameter) with six plants per basin. The trial was repeated three times.

Approximately 400 uniformly seeds of wheat and 900 of faba bean were disinfected and germinated as previously described by Yang et al. [2]. In brief, the seeds were kept fully moist until they had grown to the three-leaf stage in wheat and the six true-leaf stage in faba bean. In the monocropping systems, there were six faba bean plants per plastic basin (25 cm upper diameter, 16 cm height and 13 cm lower diameter). In the intercropping systems, three faba bean and three wheat seeds were sowed in each plastic basin in 2 L of Hoagland nutrient solution [38, 39], and the planting method was adopted as described by Yang et al. [33]. Based on the different treatments, the various concentrations of cinnamic acid and 1 mL of the spore suspension (1 × 10^6^ CFU·mL^− 1^) were simultaneously added to the base of plants [2, 4]. The nutrient solution was replaced and *F. commune* was reinoculated every 2 days [40], and an oxygenation pump was used for 24 h. All the wheat and faba bean plants were grown under natural light at 26ºC/19℃ day/night and maintained at 70–85% relative humidity.

### Evaluation of the growth of faba bean and the development of Fusarium wilt

Three faba bean seedlings from four to six fully expanded leaves were randomly chosen to measure the maximum leaf width and length in each treatment.

The presence of wilt was determined, and from each treatment 15 faba beans were selected, 45 days post-transplantation. The degree of Fusarium wilt was classified as previously described [33], as follows, Grade 0: asymptomatic; Grade 1: slight discoloration or local lesions of the root or stem base (except for the main root); Grade 2: stem bases that were not contiguous or diseased spots on the main lateral root; Grade 3: 1/3 ∼ 1/2 of the root or stem base appeared to have diseased spots, discoloration, or rot, and there were significantly fewer lateral roots; Grade 4: the stem base was surrounded by lesions, or most of the roots were discolored and rotten; and Grade 5: the plants withered and died. The development of disease was calculated as follows:

1 $$\begin{gathered}Incidence\, = \hfill \\\,\,\,\,\,\,\,\,\,number\,of\,infected\,plants/total\,number\,of\,plants \hfill \\\,\,\,\,\,\,\,\, \times 100\% \hfill \\ \end{gathered}$$

2 $$\begin{gathered}Disease\,index\, = \hfill \\\Sigma \left( \begin{gathered}number\,of\,diseased\,plants\,at\,all\,levels\, \hfill \\\times \,corresponding\,gradevalue \hfill \\ \end{gathered} \right) \hfill \\/\left( \begin{gathered}highest\,value\, \hfill \\\times \,total\,number\,of\,plants \hfill \\ \end{gathered} \right) \times 100\% \hfill \\ \end{gathered}$$

### Measurement of the absorption of nutrients by the faba bean leaves

A total of 0.1 g four to six fully expanded leaf samples (for each replicate, all plants from the 5 basins per treatment were pooled, 30 plants total) were digested using H_2_SO_4_ and hydrogen peroxide (H_2_O_2_) in digestion tubes [41]. The total nitrogen (TN) was measured from the plant filtrate using the Kjeldahl method as described by Jackson [42]. The total potassium (TK) was measured using flame photometry [43]. The total phosphorus (TP) was measured as described by Ashraf et al. [44]. A total of 0.3 g of dried leaf samples were placed in a 100 mL decoction tube, and 15 mL of nitric acid: perchloric acid (4:1v/v) was added first at low temperature (approximately 160 ℃) in a constant temperature digestion furnace until the solution turned brownish black. A volume of 10 mL of the acid mixture was added until white smoke was emitted. The solution was then boiled at 250 ∼ 300 ℃ for 1 ∼ 2 h until the solution was bright and slightly yellow. It was removed, cooled, transferred to a 25 mL volumeter bottle and finally filtered. The filtered solution was used to determine the contents of Zn, Fe and Mn by atomic absorption spectroscopy on a PerkinElmer atomic absorption spectrometer 3300 (PerkinElmer, Danbury, CT, USA). A volume of 10 mL of the filtered solution was added to a 50 mL volumetric bottle, and 50 g L^-1^ of strontium chloride was added. The content of Mg was determined by atomic absorption spectroscopy as described above.

### Determination of the photosynthetic gas exchange parameters of the faba bean leaves

The transpiration rate (Ti, mmol H2O m^− 2^ s ^− 1^), net photosynthetic rate (Pn, µmol CO2 m^− 2^ s ^− 1^), stomatal conductance (Gs, mol H2O m^− 2^ s ^− 1^) and intercellular carbon dioxide concentration (Ci, µmol⋅mol^–1^) were measured using a Li-Cor 6400 portable photosynthesis system (LI-COR, Lincoln, NE, USA). All the measurements were recorded between 09:00 and 12:00 at a light intensity of 1,200 µmol∙m^− 2^∙s^− 1^, relative humidity of 50% and temperature of 25 ℃. Four to six fully expanded leaves without any apparent fungal infection were selected from the tip of faba bean stems and placed in the Li-Cor6400 portable photosynthesis system to record the data. The measurements were recorded once the instrument had started displaying a stable reading as described by Yang et al. [4].

### Determination of the contents of photosynthetic pigments in the faba bean leaves

The contents of carotenoids, chlorophyll a, and chlorophyll b were measured using assay kits according to the manufacturer’s instructions (Sinobestbio, Shanghai, China).

### Determination of the chlorophyll fluorescence parameters in the faba bean leaves

A DualPAM-100 measurement system (Heinz Walz, Effeltrich, Germany) was used as described by Yang et al. [4] and Kramer et al. [45]. The chlorophyll fluorescence parameters were collected from 22:00 to 24:00 to determine the maximum fluorescence (Fm) and initial fluorescence (Fo), which provided the variable fluorescence Fv = (FM-Fo). The chlorophyll minimum fluorescence (Fo′), chlorophyll maximum fluorescence (Fm′) and chlorophyll stable fluorescence (Fs) after light adaptation were measured in an artificial climate chamber. The fluorescence parameters [46–48] were calculated as follows: photochemical quenching coefficient (3), maximum quantum efficiency of PSII (4), potential activity of the PSII reaction center (5), non-photochemical quenching coefficient (6), and the actual photochemical quantum yield of PSII (7).

3 $$qP = (Fm' - Fs)/(Fm' - F0')$$

4 $$Fv/Fm\, = \,(Fm-Fo)/Fm$$

5 $$Fv/Fo\, = \,(Fm-Fo)/Fo$$

6 $$NPQ = (Fm - Fm')/Fm'$$

7 $$\Phi PSII = (Fm' - Fs)/Fm'$$

### Determination of the activities of the photosynthetic enzymes in the faba bean leaves

The activities of ribulose bisphosphate carboxylase (Rubisco), ribulose bisphosphate carboxylase activator (RCA), fructose-1,6-bisphosptase (FBPase), sucrose synthase, fructose-1,6-bisphosphate aldolase (FBA), and H^+^-ATPase were measured using assay kits according to the manufacturer’s instructions (Sinobestbio).

### Determination of the contents of photosynthetic assimilates in the faba bean leaves

The contents of starch, soluble sugar and sucrose were measured using assay kits according to the manufacturer’s instructions (Sinobestbio).

### Statistical analysis

Each dataset was tested, and a normal probability plot was used to determine the variance homogeneity in SPSS 18.0 (SPSS, Inc., Chicago, IL, USA). A multi-factor analysis of variance (ANOVA) was used to analyze the data and ensure that the treatments did not interact. Least significant difference (LSD) tests were utilized, and *p* ≤ 0.05 was considered to be statistically significant. The data are presented as the averages ± standard deviation by three biological replicates.

## Results

### Influence of intercropping on the occurrence of Fusarium wilt and the growth of faba bean following inoculation with *F. commune* and cinnamic acid stress

**Fig. 1:**
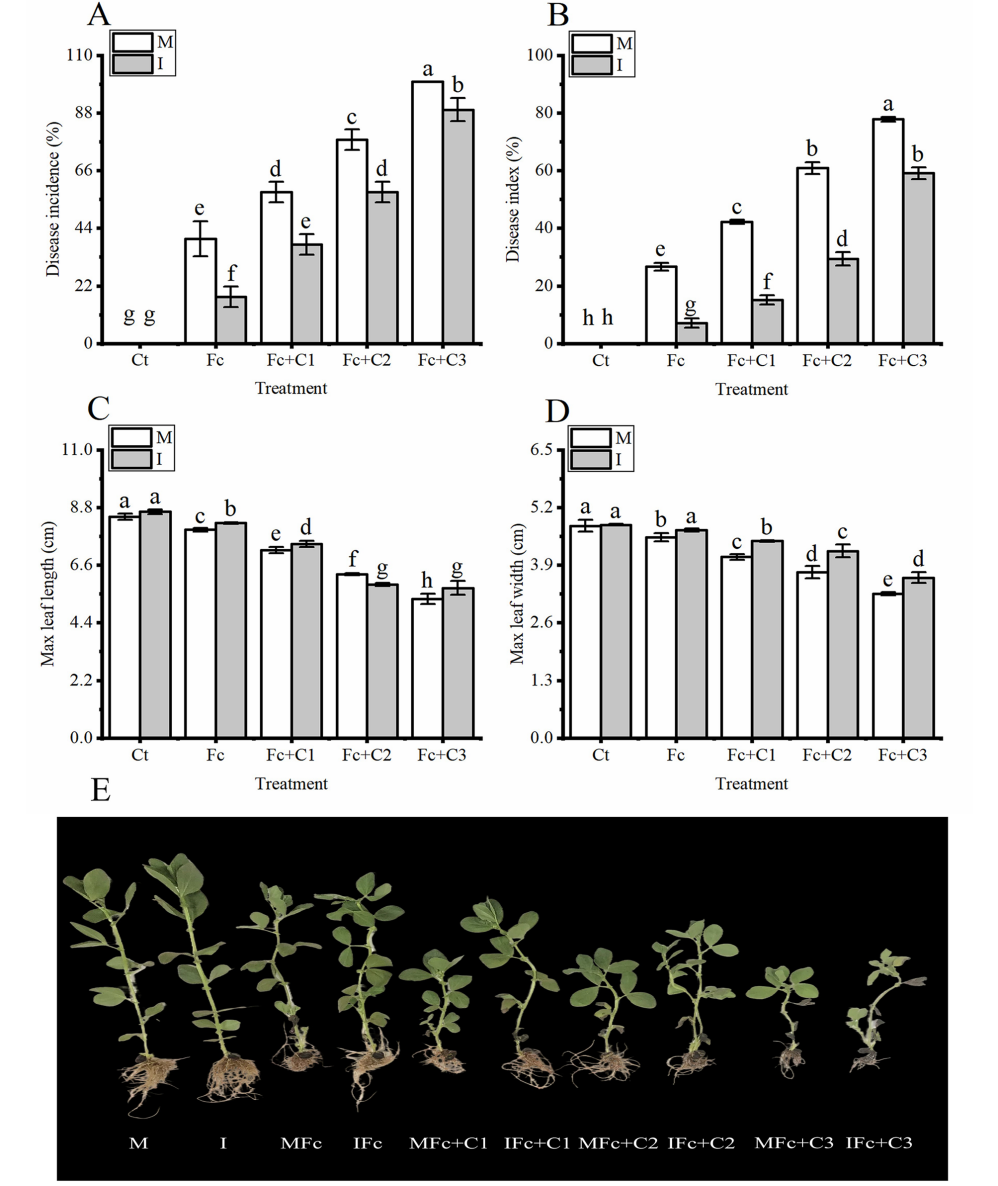
Influence of intercropping on the occurrence of Fusarium wilt in faba bean plants and the growth in response to *Fusarium commune* and cinnamic acid stress. **A**: Incidence. **B**: Disease index. **C**: Length of maximum leaf. **D**: Width of maximum leaf. **E**: The images of the symptoms on the infection under the different treatments. The values are the mean ± SD of three biological replicates. Different lowercase letters indicate a significant difference

Under the conditions of faba bean monocropping (M), the incidence and disease index in faba bean were higher in the Fc treatment (*p* < 0.05) compared with the Ct (Fig. 1A and B). Moreover, compared with the Fc treatment, the incidence and disease index were higher in the Fc + C1, Fc + C2, and Fc + C3 treatments (*p* < 0.05) (Fig. 1A and B).

The incidence and disease index were lower in the intercropping (I) faba bean and wheat compared with the faba bean monocropping (*p* < 0.05) in the treatments of Fc, Fc + C1, Fc + C2, and Fc + C3 (Fig. 1A and B).

The Fc treatment statistically significant decreased the maximum length and width of the faba bean leaves (*p* < 0.05) when the plants were grown as a monoculture compared with the Ct treatment (Fig. 1C and D). Moreover, compared with the Fc treatment, the maximum leaf length and width statistically significant decreased in the Fc + C1, Fc + C2, and Fc + C3 treatments (*p* < 0.05) (Fig. 1C and D).

The maximum leaf length and width statistically significant increased in intercropping with faba bean and wheat (*p* < 0.05) in the treatments of Fc, Fc + C1, Fc + C2, and Fc + C3 compared with the faba bean monocropping (Fig. 1C and D).

### Influence of intercropping on the photosynthetic gas exchange parameters in the faba bean leaves under *F. commune* and cinnamic acid stress

**Fig. 2:**
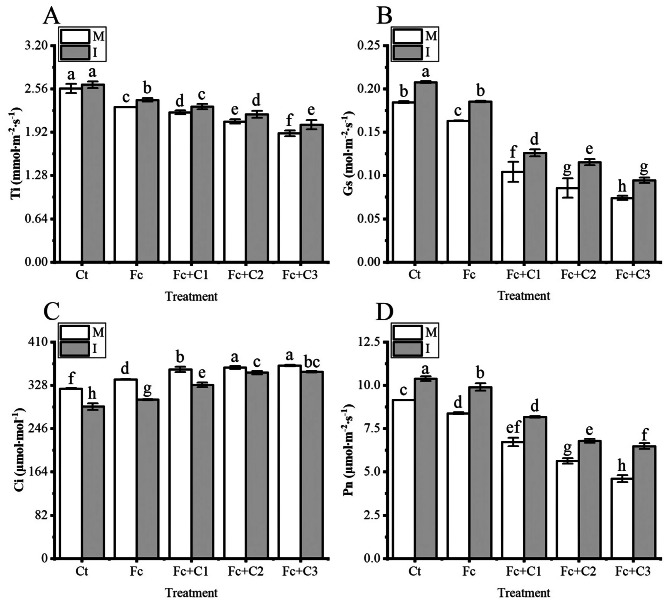
Influence of intercropping on the photosynthetic gas exchange parameters in faba bean leaves following inoculation with *Fusarium commune* and cinnamic acid stress. **A**: transpiration rate (Ti). **B**: stomatal conductance (Gs). **C**: intercellular carbon dioxide concentration (Ci). **D**: net photosynthetic rate (Pn). I, intercropping; M, monocropping. The values are the mean ± SD from three biological replicates. Different lowercase letters indicate a significant difference

The Fc treatment statistically significant decreased the Ti, Gs and Pn in the faba bean leaves and increased the Ci (*p* < 0.05) under the conditions of faba bean monocropping compared with the Ct treatment (Fig. 2A–D). Moreover, compared with the Fc treatment, the Fc + C1, Fc + C2, and Fc + C3 treatments further statistically significant decreased the Ti, Gs, and Pn, and increased the Ci (*p* < 0.05) (Fig. 2A–D).

There was a higher Ti in the faba bean leaves in the intercropping of faba bean and wheat (*p* < 0.05) under the Fc, Fc + C1, Fc + C2, and Fc + C3 conditions compared with the faba bean monocropping (Fig. 2A). The Gs and Pn in the faba bean leaves statistically significant increased and decreased the Ci when the faba bean and wheat were intercropped (*p* < 0.05) under the Ct, Fc, Fc + C1, Fc + C2, and Fc + C3 treatments compared with the monocropping of faba bean (Fig. 2B–D).

### Influence of intercropping on the absorption of nutrients in the faba bean leaves under *F. commune* and cinnamic acid stress

**Fig. 3:**
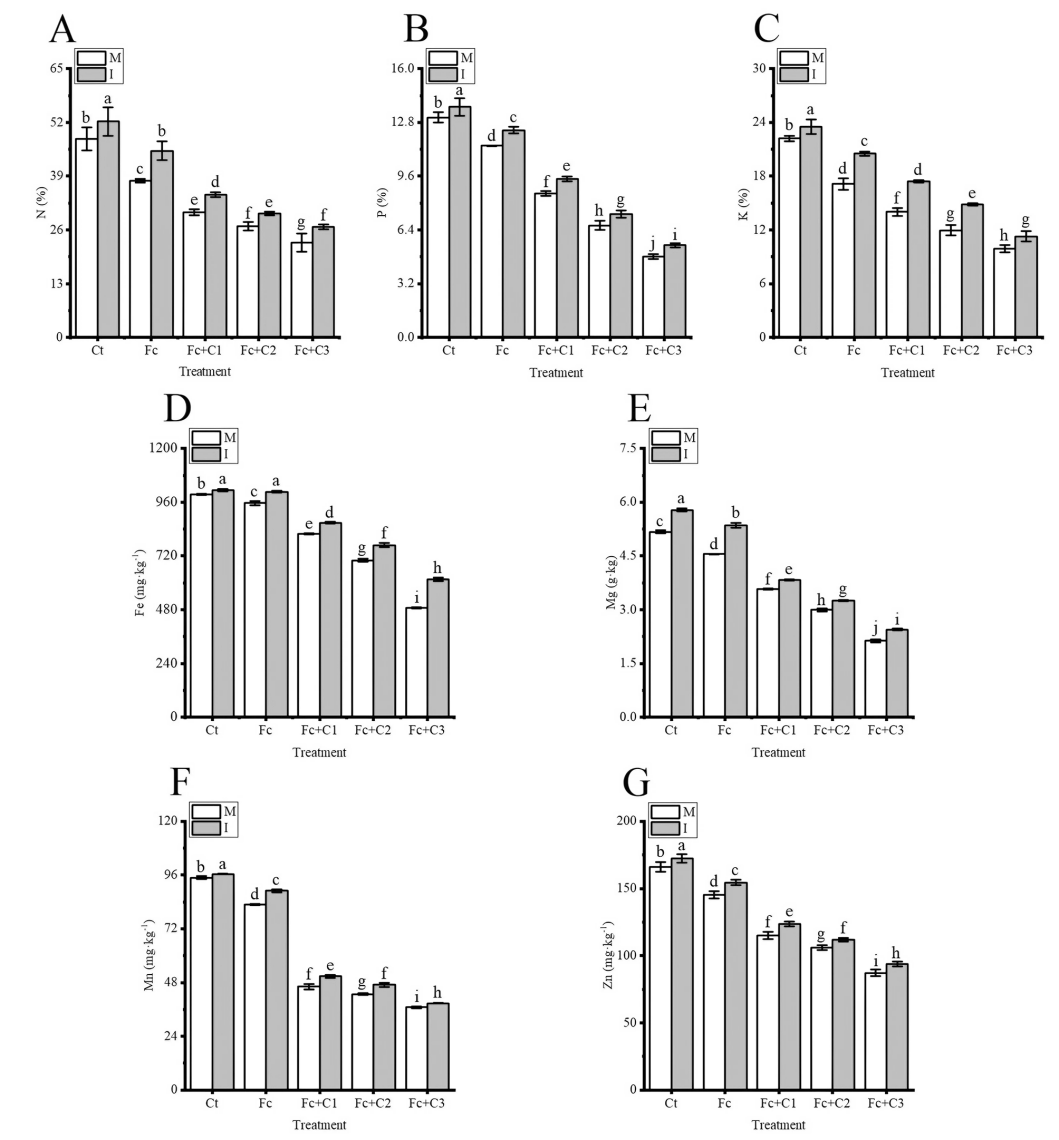
Influence of intercropping on the absorption of nutrients in faba bean leaves following inoculation with *Fusarium commune* and cinnamic acid stress. **A**: Total nitrogen (N). **B**: Total phosphorus (P). **C**: Total potassium (K). **D**: Iron (Fe). **E**: Magnesium (Mg). **F**: manganese (Mn). **G**: Zinc (Zn). Values are the mean ± SD from three biological replicates. Different lowercase letters indicate a significant difference. I, intercropping; M, monocropping

There were lower contents of total nitrogen (N), phosphorus (P), potassium (K), iron (Fe), magnesium (Mg), manganese Mn, and zinc (Zn) in the faba bean leaves in the Fc treatment (*p* < 0.05) under the condition of faba bean monocropping compared with the Ct (Fig. 3). Moreover, the Fc + C1, Fc + C2, and Fc + C3 treatments statistically significant reduced the contents of N, P, K, Fe, Mg, Mn, and Zn even more in the faba bean leaves compared with the Fc treatment (*p* < 0.05) (Fig. 3).

The contents of N, P, K, Fe, Mg, Mn, and Zn in the faba bean leaves statistically significant increased when the faba bean and wheat were intercropped under the Ct, Fc, Fc + C1, Fc + C2, and Fc + C3 conditions compared with faba bean monocropping (*p* < 0.05) (Fig. 3).

### Influence of intercropping on the photosynthetic pigments in faba bean leaves under *F. commune* and cinnamic acid stress

**Fig. 4:**
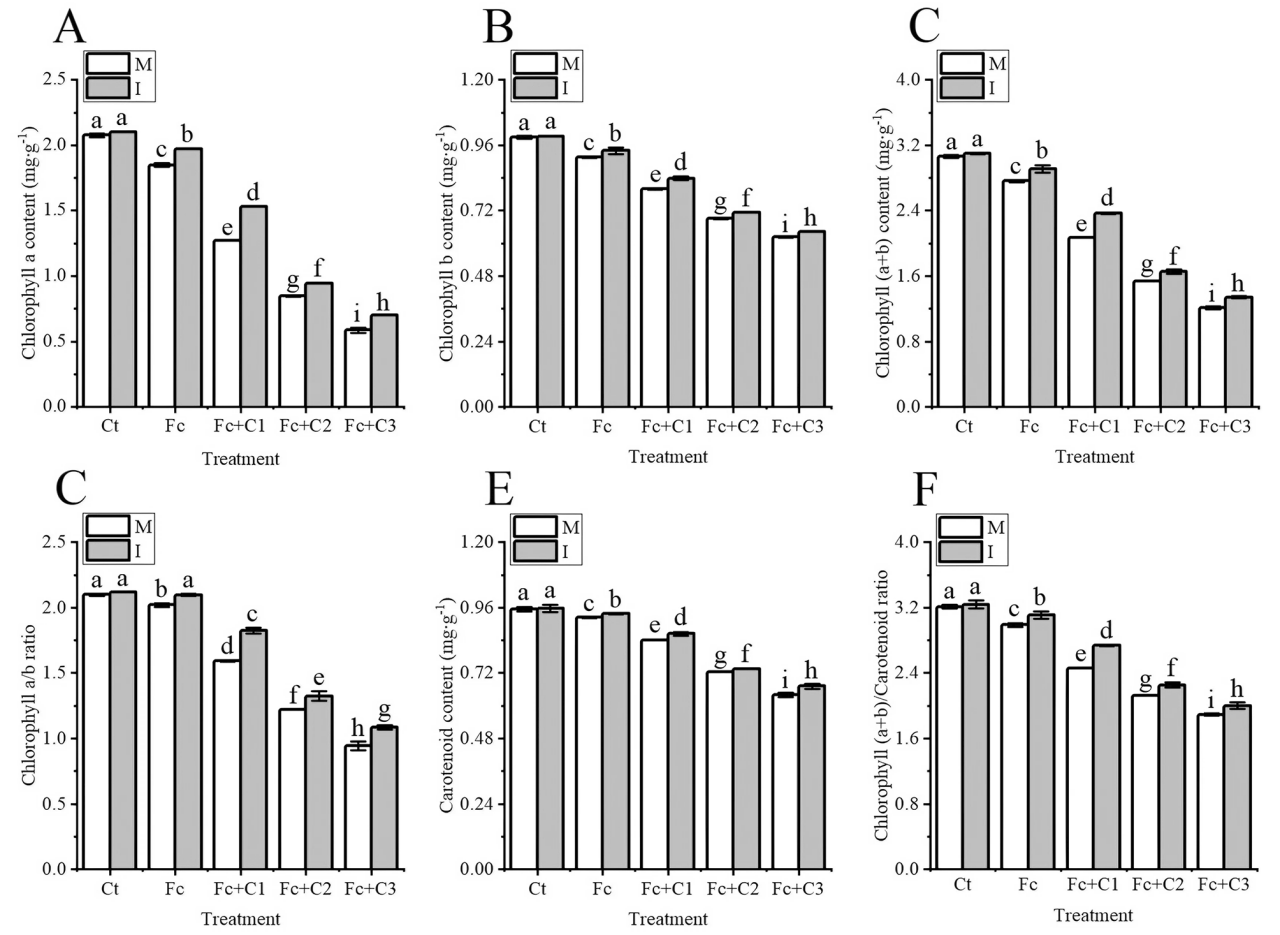
Influence of intercropping on the photosynthetic pigments in faba bean leaves following infection with *Fusarium commune* and cinnamic acid stress. **A**: Chlorophyll (a) **B**: Chlorophyll (b) **C**: Chlorophyll a + b. **D**: Chlorophyll a/b ratio **E**: Carotenoids. **F**: Chlorophyll (a + b)/ Carotenoid. Values are the mean ± SD from three biological replicates. Different lowercase letters indicate a significant difference. I, intercropping; M, monocropping

The contents of chlorophyll a, b, a + b and carotenoids in the faba bean leave were lower in the Fc treatment compared with the Ct (*p* < 0.05) under the conditions of faba bean monocropping (Fig. 4A, B, C and E). The chlorophyll a/b ratio and chlorophyll (a + b)/carotenoid ratio also decreased (*p* < 0.05) (Fig. 4D and F).

The contents of chlorophyll a, b, and the ratio of chlorophyll a/b and chlorophyll (a + b)/carotenoid in the leaves of faba bean statistically significant increased during the intercropping of faba bean and wheat (*p* < 0.05) under the Fc, Fc + C1, Fc + C2, and Fc + C3 conditions compared with the faba bean monocropping (Fig. 4).

### Influence of intercropping on the chlorophyll fluorescence parameters in the faba bean leaves under *F. commune* and cinnamic acid stress

**Fig. 5:**
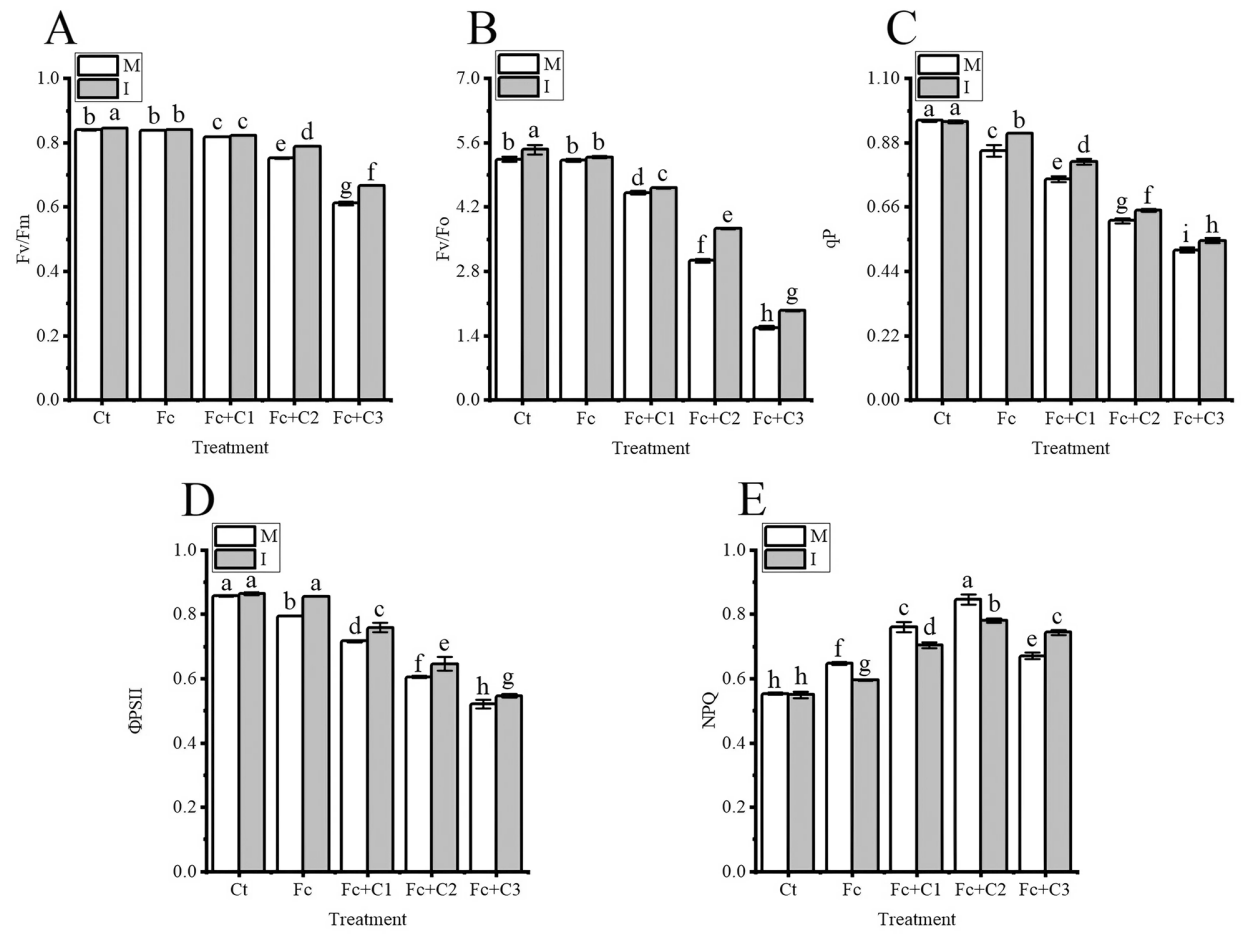
Influence of intercropping on the chlorophyll fluorescence parameters in the faba bean leaves in response to *Fusarium commune* and cinnamic acid stress. **A**: Maximal quantum efficiency of PSII (Fv/Fm). **B**: Potential activity of the PSII reaction center (Fv/Fo). **C**: Photochemical quenching coefficient (qP). **D**: Actual photochemical quantum yield of PSII (ФPSII). **E**: Non-Photochemical quenching coefficient (NPQ). The values are the mean ± SD of three biological replicates; Different lowercase letters indicate a significant difference. I, intercropping; M, monocropping; PSII, Photosystem II.

The photochemical quenching coefficient (qP) and actual photochemical quantum yield of PSII (ФPSII) were lower, and the non-photochemical quenching coefficient (NPQ) in the faba bean leaves was higher in the Fc treatment (*p* < 0.05) under the condition of faba bean monocropping compared with the Ct treatment (Fig. 5C and D). The Fc + C1, Fc + C2, and Fc + C3 treatments statistically significant reduced the maximal quantum efficiency of PSII (Fv/Fm), potential activity of the PSII reaction center (Fv/Fo), ФPSII and photochemical quenching coefficient (qP) and statistically significant increased the NPQ in the faba bean leaves compared with the Fc treatment (*p* < 0.05) (Fig. 5).

The Fv/Fm in faba bean leaves statistically significant increased in intercropping with faba bean and wheat (*p* < 0.05) under the Fc + C2, and Fc + C3 conditions compared with the faba bean monocropping (Fig. 5A). The Fv/Fo statistically significant increased in the faba bean leaves during intercropping with faba bean and wheat (*p* < 0.05) under the Fc + C1, Fc + C2, and Fc + C3 conditions compared with the faba bean monocropping (Fig. 5B). The qP and ФPSII in the faba bean leaves statistically significant increased during intercropping with faba bean and wheat (*p* < 0.05) under the Fc, Fc + C1, Fc + C2, and Fc + C3 conditions compared with monocropping (Fig. 5C and D). The NPQ in faba bean leaves was lower during the intercropping of faba bean and wheat under the Fc, Fc + C1, and Fc + C2 conditions. However, the NPQ statistically significant increased during intercropping with wheat and faba bean compared with monocropping under the Fc + C3 conditions (*p* < 0.05) (Fig. 5E).

### Influence of intercropping on the photosynthetic enzymes in faba bean leaves under *F. commune* and cinnamic acid stress

**Fig. 6:**
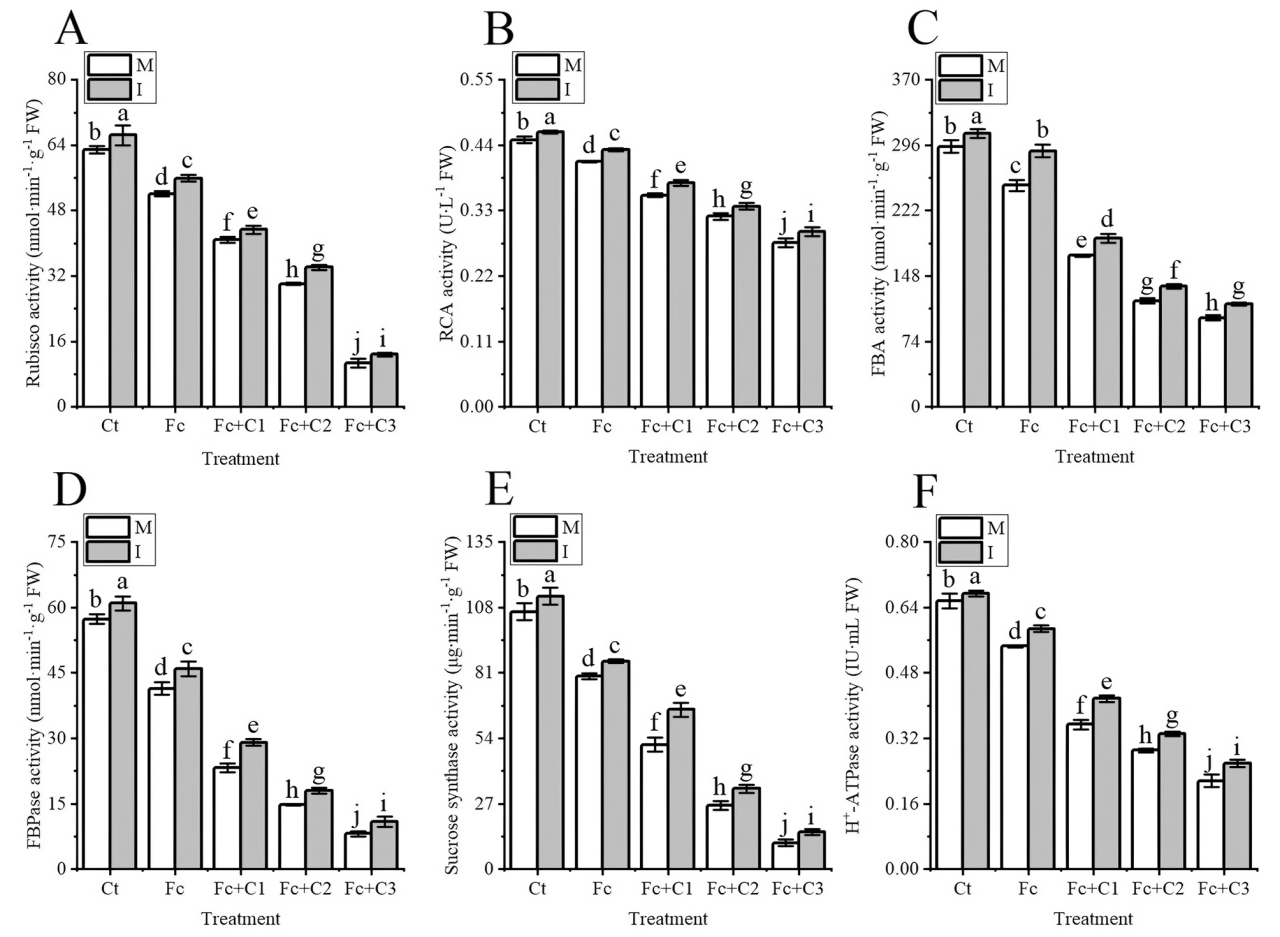
Influence of intercropping on the activities of photosynthetic enzymes in faba bean leaves under *Fusarium commune* and cinnamic acid stress. **A**: Ribulose bisphosphate carboxylase (Rubisco). **B**: Ribulose bisphosphate carboxylase activator (RCA). **C**: Fructose-1,6-bisphosphate aldolase (FBA). **D**: Fructose-1,6-bisphosptase (FBPase). **E**: Sucrose synthase. **F**: H^+^-ATP. Values are the mean ± SD of three biological replicates. Different lowercase letters indicate a significant difference. I, intercropping; M, monocropping

The Fc treatment statistically significant decreased the activities of ribulose bisphosphate carboxylase (Rubisco), ribulose bisphosphate carboxylase activator (RCA), fructose-1,6-bisphosphate aldolase (FBA), fructose-1,6-bisphosptase (FBPase), sucrose synthase, and H^+^-ATPase in faba bean leaves compared with the Ct treatment under faba bean monocropping conditions (*p* < 0.05) (Fig. 6). In addition, the activities of Rubisco, RCA, FBA, FBPase, sucrose synthase, and H^+^-ATP in the faba beans were significantly reduced even further in the Fc + C1, Fc + C2, and Fc + C3 treatments compared with the Fc treatment (*p* < 0.05) (Fig. 6).

The activities of Rubisco, RCA, FBA, FBPase, sucrose synthase, and H^+^-ATP in the faba bean leaves were higher during intercropping with wheat and faba bean (*p* < 0.05) under the Ct, Fc, Fc + C1, Fc + C2, and Fc + C3 conditions compared with faba bean monocropping (Fig. 6).

### Influence of intercropping on the photosynthetic assimilates in the faba bean leaves under *F. commune* and cinnamic acid stress

**Fig. 7:**
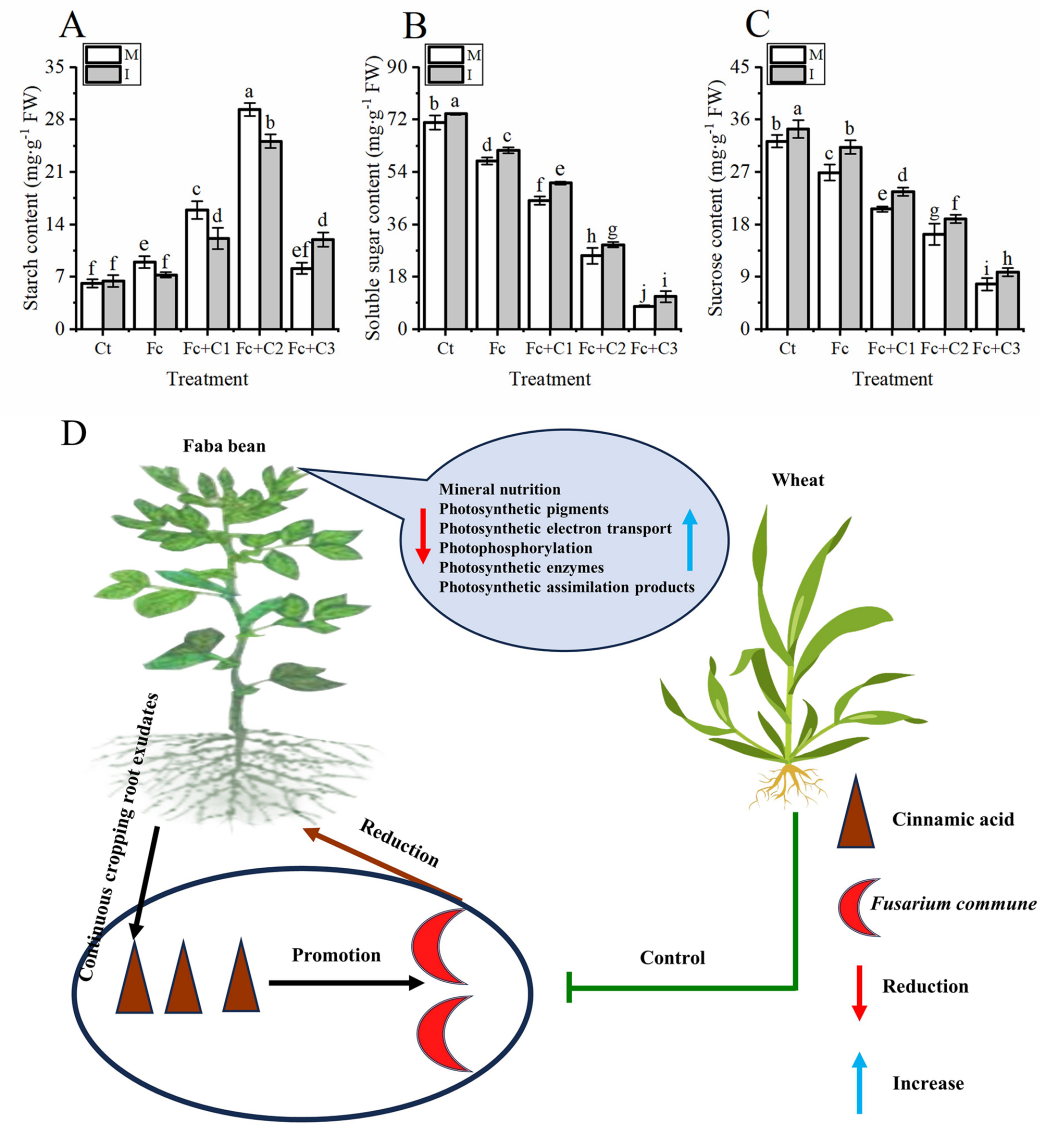
Influence of intercropping on the photosynthetic assimilates in faba bean leaves in response to *Fusarium commune* and cinnamic acid stress. **A**: Starch. **B**: Soluble sugar. **C**: sucrose. **D**: Conceptual model of wheat and faba bean intercropping facilitating photosynthesis in the faba beans reduced the amount of Fusarium wilt to respond to *Fusarium commune* and cinnamic acid stress. Values are the mean ± SD of three biological replicates. Different lowercase letters indicate a significant difference

The Fc treatment statistically significant increased the content of starch but decreased soluble sugar and sucrose content in the faba bean leaves (*p* < 0.05) under monocropping conditions compared with the Ct (Fig. 7). The Fc + C1 and Fc + C2 treatments statistically significant increased the content of starch in the faba bean leaves compared with the Fc treatment (*p* < 0.05) (Fig. 7A). The Fc + C1, Fc + C2, and Fc + C3 treatments statistically significant decreased soluble sugar and sucrose content in the faba bean leaves compared with the Fc treatment (*p* < 0.05) (Fig. 7B and C).

Under the Fc + C1, and Fc + C2 conditions compared with faba bean monocropping, there was less starch in the faba bean leaves during the intercropping of faba bean and wheat, but there was more starch during the intercropping of wheat and faba bean (*p* < 0.05) under the Fc + C3 condition compared with the faba bean monocropping (Fig. 7A). There were higher contents of soluble sugar and sucrose during intercropping with faba bean and wheat (*p* < 0.05) under the Ct, Fc + C1, Fc + C2, and Fc + C3 conditions compared with the faba bean monocropping (Fig. 7B and C).

## Discussion

Soilborne pathogens are considered to be the primary cause of soil diseases, which is true for most crops [49]. Previous studies have often reported that soilborne pathogenic fungi can reduce the growth of plants and their yields [50, 51]. The findings of this study are consistent with these conclusions. The faba bean leaves grew less following inoculation with *F. commune* compared with those of the plants that had not been inoculated with *F. commune* (Fig. 1C and D). It is worth noting that *Fusarium* can survive in the soil for many years [4, 52]. However, allelopathic autotoxins can exude from the plant roots into the rhizosphere [9]. Cucumber leaves grew less, and the severity of Fusarium wilt was higher following inoculation with *F. oxysporum* f. sp. *cucumerinum* and cinnamic acid compared with plants that had not been inoculated with *F. oxysporum* f. sp. *cucumerinum* [11]. Similar results were obtained in this study (Fig. 1), which showed that the combined effect of *F. commune* and cinnamic acid reduced the growth of faba beans and stimulated the development of Fusarium wilt. Appropriate amounts of intercropping promote the growth of plants and control diseases. There were a lower incidence and disease index of red crown rot caused by *Calonectria illicicola* and were longer roots in soybean during intercropping with maize and soybean compared with monocropped soybean [21]. There were similar results in this study, and intercropping with wheat and faba bean significantly reduced the occurrence of Fusarium wilt and increased leaf growth compared with faba bean monocropping following inoculation with *F. commune* and cinnamic acid stress (Fig. 1). The results showed that the occurrence of faba bean Fusarium wilt was effectively reduced by faba bean and wheat intercropping.

Photosynthesis is a basic biosynthetic process for plants to obtain energy and organic matter for growth and development. Inoculation with *F. oxysporum* f. sp. *cucumerinum* and the addition of cinnamic acid significantly reduced the Pn and Gs of cucumber leaves and increased the Ci compared with the sole inoculation with *F. oxysporum* f. sp. *cucumerinum* [11]. Similar results were obtained in this study (Fig. 2). This may be owing to the reduction of these parameters through non-stomatal or stomatal limiting factors [53]. Some studies have shown that under stress, the reduction of Gs can reduce the Ci and Pn, which indicates that stomatal factors were the reasons for these changes [53]. However, in this study, the Ci was enhanced and the G*s* reduced, which resulted in changes owing to non-stomatal factors; instead, they were most likely owing to chloroplast damage [54]. Previous studies found that *Fusarium* primarily destroyed the host vascular bundle tissue system, which is responsible for the transport of water and nutrients [4]. A decrease in the Ti can affect the ability of plants to absorb water and nutrients. Among them, nutrient mineral elements play a vital role in the survival of plants and their photosynthesis [55, 56]. Compared with soybean monocropping, the intercropping of flax (*Linum usitatissimum*) with soybean significantly increased the Pn, Gs and Ti and decreased the Ci [57]. In this study following inoculation with *F. commune* and cinnamic acid stress, the Pn, Gs and Ti of the faba bean leaves were higher, and the Ci was lower during the intercropping of wheat and faba bean compared with the monocropping of faba bean (Fig. 2).

Chloroplasts are the primary organelles of photosynthesis, and the photosynthetic pigments have a vital role in the absorption, transfer and capture light energy [58, 59]. N and Mg, as components of chlorophyll, and Fe, Mn and Zn play important roles in the biosynthesis of chlorophyll [60–62]. In this study, *F. commune* + cinnamic acid significantly reduced the contents of chlorophyll a, chlorophyll b, (a + b) and carotenoids and the chlorophyll (a/b) ratio compared with the inoculation of *F. commune* (Fig. 4). The contents of photosynthetic pigments also affect electron transport by the photosynthetic electron transporters [63]. The contents of carotenoids, chlorophyll a, and chlorophyll b in pakchoi (*Brassica rapa* subsp. *chinensis*) leaves were higher during the intercropping of pakchoi and lettuce (*Lactuca sativa*) compared with pakchoi monocropping [64]. In this study, under *F. commune* and cinnamic acid stress, the intercropping of faba bean and wheat significantly increased the contents of chlorophyll a, chlorophyll b, (a + b) and carotenoids and the chlorophyll (a/b) ratio compared with faba bean monocropping (Fig. 4). Photosynthetic pigments in the plant chloroplasts absorb light and transfer their energy to PS II, and part of the light energy is re-emitted, which is known as chlorophyll fluorescence [65, 66]. When the PSII reaction center is fully open, the intensity of fluorescence emission reaches its minimum (Fo) and when the reaction center is completely closed, the intensity of fluorescence emission reaches its maximum (Fm) [67–69]. Where Fv is variable fluorescence, Fv/Fo and Fv/Fm are relatively stable under certain conditions. Ye et al. [11] reported that the Fv/Fm in the cucumber leaves was lower in *F. oxysporum* f. sp. *cucumerinum* + cinnamic acid compared with the sole inoculation with *F. oxysporum* f. sp. *cucumerinum*. In this study, *F. commune* + cinnamic acid significantly reduced the Fv/Fm and Fv/Fo in the faba bean leaves compared with inoculation with *F. commune* (Fig. 5A and B). The decrease of Fv/Fm and Fv/Fo indicated that the combination of *F. commune* with cinnamic acid could reduce the rate and efficiency of the conversion of primary light energy into chemical energy, which can result in the insufficient assimilation of energy from photosynthetic carbon. qP is closely contacted to the redox potential of electron acceptor plastoquinone QA (PSII reaction center). A larger qP leads to the oxidation of QA, which results in a more open PSII reaction center. In contrast, a smaller qP leads to a reduction in QA, and the PSII reaction center becomes less open [70]. Ye et al. [11] reported that the qP in cucumber leaves was lower in treatments of *F. oxysporum* f. sp. *cucumerinum* + cinnamic acid compared with inoculation with *F. oxysporum* f. sp. *cucumerinum* alone. There were similar results in this study, and *F. commune* + cinnamic acid significantly reduced Fe and Mn compared with the inoculation of *F. commune* (Figs. 3D and F and 5C). Mn plays an important role in the oxygen-evolving complex, and Fe plays a vital role in electron transport [71, 72]. The higher closure of the PSII reaction center will result in an inability to complete a stable charge separation, which results in the failure of the linear transfer of photosynthetic electrons. This can lead to a decrease in the photophosphorylation of ATP. The ФPSII represents the PSII electron transport quantum yield in the optical system. Electron transport is always coupled to the photophosphorylation of ATP. Ye et al. [11] reported that the ФPSII in cucumber leaves was lower in the *F. oxysporum* f. sp. *cucumerinum* + cinnamic acid treatment compared with *F. oxysporum* f. sp. *cucumerinum* treatment alone. Similar results were observed in this study, and *F. commune* + cinnamic acid significantly reduced P in the faba bean leaves compared with the inoculation of *F. commune* (Figs. 3B and 5D). Among them, P plays a crucial role in photophosphorylation [73]. This reduction in quantum yield will also reduce the amount of photophosphorylated ATP. However, the photosystem can consume the excess light energy absorbed by PSII by increasing the dissipation of non-radiative heat [74, 75]. Ye et al. [11] reported that the NPQ in cucumber leaves was higher in the *F. oxysporum* f. sp. *cucumerinum* + cinnamic acid treatment compared with the leaves that were inoculated with *F. oxysporum* f. sp. *cucumerinum* alone. Similar results were obtained in this study (Fig. 5E). The increase in NPQ forces the host to release the energy absorbed by PSII through non-radiative heat dissipation, but this may prevent the host from fully utilizing the captured light energy. The results showed that the combination of *F. commune* and cinnamic acid resulted in the absorption of light energy and obstruction of the electron transport chain, particularly in chloroplast photoreaction center II. The potential activity of chloroplast PSII is reduced; the ability to convert light energy to chemical energy is impaired; the quantum transfer rate is decreased, and the amount of photophosphorylation is reduced, which may affect the assimilation of photosynthetic carbon in the host. Intercropping with maize and peanut (*Arachis hypogeae*) significantly increased the peanut Fv/Fm, qP, and ФPSII and decreased the NPQ compared with peanut monocropping [76]. The Fv/Fo, Fv/Fm, qP, and ФPSII and the absorption of nutrients were higher in the faba bean leaves grew under *F. commune* and cinnamic acid stress compared with faba bean monocropping, and the NPQ was lower in wheat -faba bean intercropping (Figs. 3 and 5). Simultaneously, the NPQ of was significantly increase in wheat and faba bean intercropping under *F. commune* and 200 mg·L^− 1^ cinnamic acid stress. It is possible that the chloroplasts of faba bean have been seriously damaged from *F. commune* and 200 mg·L^− 1^ cinnamic acid stress in monocropping system. Some studies have shown that the exudates of wheat roots could inhibit the action of *Fusarium oxysporum* f. sp. *niveum* when watermelon and wheat were intercropped [77]. It is possible that faba bean sought help from wheat in response to *F. commune* and cinnamic acid stress to release the energy absorbed by PSII through non-radiative heat dissipation to alleviate the photoinhibition of photosynthesis. The results indicated that intercropping with faba bean and wheat promoted the absorption of the light energy and the conversion of light energy to chemical energy, promoted the transfer of electrons, and may increase the amount of photophosphorylation.

The binding of CO_2_ to ribulose diphosphate (RuDP) is the first key reaction in the dark reaction Calvin cycle, which is then catalyzed by Rubisco to produce glyceraldehyde-3-phosphate. Rubisco directly affects the rate of assimilation of CO_2_. The activity of RCA affected the efficiency of carboxylation and the degree of activation of Rubisco [78, 79]. There were lower activities of Rubisco and RCA in the cucumber leaves due to exogenous application of cinnamic acid compared with the control [80]. The activities of Rubisco and RCA in the faba bean leaves and the content of Mg were lower in the *F. commune* + cinnamic acid treatment compared to the sole inoculation with *F. commune* (Fig. 6A and B). Mg plays a vital role in the activation of photosynthetic enzymes, such as Rubisco and FBPase [81, 82]. Corn (*Zea mays*) and peanut intercropping significantly increased the activity and activation of Rubisco compared with peanut monocropping [76]. There were higher activities of Rubisco and RCA and Mg content when the wheat and faba bean were intercropped under *F. commune* and cinnamic acid stress compared with the monocropping of faba bean (Fig. 6A and B). FBA, FBPase and sucrose synthase are key rate-limiting enzymes during the process of carbon assimilation [83–85]. There were lower activities of FBA, FBPase, sucrose synthase and H^+^-ATPase in cucumber following the addition of exogenous cinnamic acid compared with the control treatment [80, 86]. The activities of FBA, FBPase, sucrose synthetase and H^+^-ATPase were significantly reduced in the *F. commune* + cinnamic acid treatment compared with the sole inoculation of *F. commune* (Fig. 6C–F). The decrease in the activities of photosynthetic enzymes may be unfavorable to the production and transport of the products of photosynthetic carbon assimilation in faba bean. There were higher activities of FBA, FBPase, sucrose synthetase and H^+^-ATPase when the wheat and faba bean were intercropped under *F. commune* and cinnamic acid stress compared with faba bean monocropping (Fig. 6C–F). Some studies have shown that the exudates of wheat roots could inhibit the action of *Fusarium oxysporum* f. sp. *niveum* when watermelon and wheat were intercropped [77]. It is possible that faba bean sought help from wheat in response to *F. commune* and cinnamic acid stress to promote the activities of photosynthetic enzymes for faba bean.

Stressed leaves are characterized by a reduction in the rates of photosynthesis, which leads to a reduction in the concentrations of soluble sugars (nonstructural carbohydrates) and usually the accumulation of starch (structural carbohydrates) [87]. In this study, there were lower levels of soluble sugar, sucrose, P and K in the faba bean leaves and higher levels of starch in the *F. commune* + cinnamic acid treatment compared with the sole inoculation of *F. commune* (Figs. 3B and C and 7). K plays a crucial role in the transformation of photosynthetic products and their transport [88]. The possible reason is that the activities of FBA, FPBase, sucrose synthetase and H^+^-APT and the contents of P and K decrease, which results in a reduction from the biosynthesis and output of nonstructural sucrose in the carbon assimilation process of faba bean, which, in turn, promotes the increase of starch biosynthesis. This results in an inability to transport the products of photosynthetic assimilation. Simultaneously, the content of structural sugar (starch) did not increase significantly in the plants treated with *F. commune* and 200 mg·L^− 1^ cinnamic acid, and the contents of nonstructural sugar (sucrose and soluble sugar) decreased significantly. It is possible that the chloroplasts of faba bean have been seriously damaged. There were higher contents of soluble sugar and sucrose in intercropping with soybean and corn compared with corn monocropping [89]. There were higher contents of soluble sugar and sucrose, P and K in the faba bean leaves under *F. commune* and cinnamic acid stress compared with the monocropping of faba bean, and a lower content of starch when the faba bean and wheat were intercropped (Figs. 3B and C and 7). The possible reason for this is that the intercropping of wheat and faba bean improves the amount of nutrients, increases the content of photosynthetic pigments in the faba bean leaves, promotes electron transfer, photophosphorylation activities, and key photosynthetic enzymes, and thus, promotes the production of photosynthetic assimilates.

The combination of autotoxic compounds with soilborne pathogens has been intensively studied because of its ability to dramatically inhibit the growth of plants and cause severe soilborne diseases [4, 86, 90]. In this study, faba bean wilt was correlated with a decrease in photosynthesis, although it was promoted during intercropping when *F. commune* infection was combined with cinnamic acid (Fig. 7D). The development of faba bean wilt from the field was observed to be higher than that in the hydroponic experiments conducted in this study (Supplementary Data Figure S3 and S4) owing to multiple factors from the complex environment. Notably, *F. commune* can survive for many years in the soil. The continuous cultivation of faba bean resulted in the accumulation of autotoxic factors that accumulated and deleteriously affected the rhizosphere soil habitat, which made it conducive to the growth of *Fusarium*; thus, enhancing the susceptibility of the faba bean plants [2, 4, 52]. These results reveal the necessity of developing a comprehensive strategy to control faba bean wilt, which includes improving the microecological environment in the rhizosphere and inhibiting the growth of pathogenic fungi. A novel method to control wilt disease should be considered based on the interactions of fungal pathogens with the rhizosphere microecosystem. We propose that the sustainable management of crop diseases is fundamental to control the greater development of soilborne diseases in agricultural ecosystems. Therefore, we combined diversified planting with intercropping to inhibit the growth of pathogenic fungi and improve the utilization of natural resources (photosynthesis) for the hosts. This model can effectively and sustainably control the development of wilt to promote sustainable production and improve photosynthesis in faba bean.

## Conclusions

The faba bean grew less following inoculation with *F. commun*e and treatment with cinnamic acid. This combination reduced the absorption of nutrients, contents of photosynthetic pigments, efficiency of electron transport, photophosphorylation and the key activities of the photosynthetic enzymes of faba bean leaves. These factors jointly inhibited photosynthesis, reduced the production of photosynthetic assimilates and the growth of faba bean, and stimulated the occurrence of Fusarium wilt. Under *F. commune* and cinnamic acid stress, faba bean- wheat intercropping increased the absorption of nutrients, contents of photosynthetic pigments, efficiency of electron transport, photophosphorylation and the key activities of the photosynthetic enzymes of faba bean leaves. These factors jointly promoted the photosynthesis of faba bean, improved the production of photosynthetic assimilates, decreased the development of Fusarium wilt and promoted the growth of faba bean. However, more research is merited to explore how intercropping promotes photosynthesis at the molecular level under combined *Fusarium* and autotoxic stress and improve the understanding of plants for their roles.

### Electronic supplementary material

Below is the link to the electronic supplementary material.

Supplementary Material 1

## Data Availability

The data that support the fndings of this study are available from the corresponding author upon reasonable request.
