# Supplementary material for: Intercropping improves faba bean photosynthesis and reduces disease caused by Fusarium commune and cinnamic acid-induced stress
Source: BMC Plant Biol. 2024 Jul 9;24:650. doi: 10.1186/s12870-024-05326-8 (PMC11232231; doi:10.1186/s12870-024-05326-8)
Supplement: Supplementary file 1 — Supplementary Material 1 [file 12870_2024_5326_MOESM1_ESM.docx]

**Materials and Methods**

**Evaluation of *F. commune* spore germination in vitro**

Assessment of spore germination in vitro was referred to Wu et al. (2009) method.

**Field Trials**

Field experiments were conducted from October 2016 to May 2017 (planted for three years), October 2018 to May 2019 (planted for five years), October 2020 to May 2021 (planted for seven years) and October 2021 to May 2022 (planted for eight years) in Asan County, Yuxi City, Yunnan Province, China (24° 11′N, 102° 24′E; 1,540 m a.s.l.). It is located in a humid subtropical region, and has been growing faba beans for seven consecutive years with a soil type of Haplic Lixisol and light loam (0 to 20 cm). The basic physicochemical traits of the soil in test site were as follows: 2016-2017, organic matter, 28.5 g·kg^-1^; available N, 106 mg·kg^-1^; available P, 33.5 mg·kg^-1^; available K, 98.5 mg·kg^-1^ and pH 6.7. 2018-2019, organic matter, 28.4 g·kg^-1^; available N, 102 mg·kg^-1^; available P, 33.2 mg·kg^-1^; available K, 96.1 mg·kg^-1^ and pH 6.48. 2020-2021, organic matter, 28.6 g·kg^-1^; available N, 98 mg·kg^-1^; available P, 31.2 mg·kg^-1^; available K, 93.4 mg·kg^-1^ and pH 6.32. 2021-2022, organic matter, 28.3 g·kg^-1^; available N, 96.1 mg·kg^-1^; available P, 30.3 mg·kg^-1^; available K, 92.9 mg·kg^-1^ and pH 6.24.

**Experimental material**

Faba bean seeds (*Vicia faba* L.) variety '89-147' and wheat (*Triticum aestivum* L.) variety 'Yunmai 53' were purchased from the Yunnan Academy of Agricultural Sciences (Kunming, China).

**Experimental design**

Faba beans (*Vicia faba* L.) and wheat (*Triticum aestivum* L.) were sown on October 25, 2021, and harvested on April 30, 2022. No pesticides or agronomic measures were used during its growth, and blight in the field occurs naturally.

The N, P, and K fertilizers used during the test were urea (N, 46%), phosphate pentoxide (P_2_O_5_,16%), and potassium sulfate (K_2_O, 50%), respectively. The application rates of nitrogen, phosphate and potassium fertilizer for monocropping and intercropping faba bean were both 90 kg·ha^-1^, and both were used as base fertilizers at the time of sowing.

The field planting patterns are shown in Figure 1. The plant spacing in the faba bean monocropping system was 0.1 m, and the row spacing was 0.3 m. In the faba bean and wheat intercropping system, the plant spacing in faba bean was 0.1 m, and the row spacing was 0.3 m. the plant spacing in wheat was 0.2 m, and the row spacing 0.3 m between the faba bean and wheat.

A total of 18 lines of faba beans were planted for the monocropping system, and two lines of faba beans alternating with six lines of wheat were alternately sown for the intercropping system. The faba beans were planted used [hill-seeding](javascript:;), and the wheat was sown in a strip.

This study used a randomized block trial design with a multi-factor arrangement with the following treatments: (1) the early stage of monocropping faba bean onset (Branch stage): M, i; (2) the peak stage of monocropping faba bean onset (Flowering stage): M, ii; (3) late stage of monocropping faba bean onset (Pod-filling stage): M, iii; (4) the early stage of intercropping faba bean onset (Branch stage): I, i; (5) the peak stage of intercropping faba bean onset (Flowering stage): I, ii; and (6) late stage of intercropping faba bean onset (Pod-filling stage): I, iii. Six treatment combinations were included, and each treatment combination was repeated three times. The two treatment plots and their four replicate plots were randomly arranged, each with a plot area of 5.4m×6m=32.4m^2^


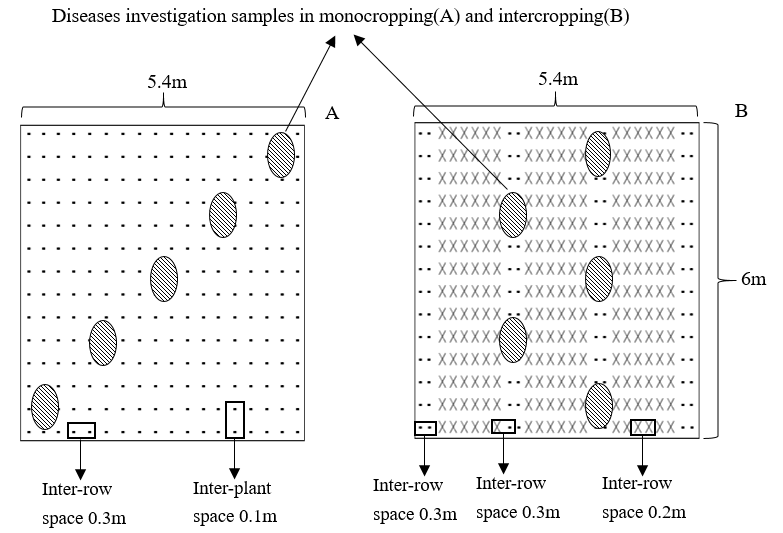


**Figure S1.** Schematic diagram of the field planting patterns of faba bean and wheat. A：Faba bean monocropping plot，B：Faba bean and wheat intercropping plot；- for faba bean plants, × for wheat plants.

[**Survey**](javascript:;) **of faba bean wilt disease**

Faba bean wilt was investigated during the three periods (early stage, peak stage and late stage of onset). Five diagonal points were randomly selected in the monocropping faba bean plots. Three plants at each point were selected for analysis, which was a total of 15 faba bean in each spot. In the faba bean and wheat intercropping plots, five points were selected on two faba bean belts. Two points were selected in the first faba bean belt, and three points were selected in the second faba bean belt. Three plants at each point were selected for analysis, which was a total of 15 faba beans in each plot. The severity of faba bean wilt was rated by the following criteria at different stages: 0: No symptoms of infection; 1: Faba bean stem bases or peripheral roots had small patches or slight discoloration; 2: Faba bean stem bases or roots had uneven damage, 3: Faba bean stem bases or roots had lesions, and approximately one-third to one-half of the stem bases or roots were discolored or wilting. In addition, the transverse root was reduced, 4: Faba bean stem bases or roots were completely discolored and withered, and 5: Faba bean plants had completely withered and died. The incidence of wilt and the disease index were calculated as follows:

Incidence = number of infected plants/total number of investigated plants ×100%

Disease index = Σ(number of diseased plants at all levels × corresponding grade value)/(highest value × total number of investigated plants)×100%.

**Results and Discussion**

**Effect on the occurrence of faba bean wilt during different onset periods and intercropping effect

**

**Figure S2**. Effect of benzoic acid on spore germination of [*Fusarium*](javascript:;) *commune.* The values were the averages ± standard deviation by three biological replicates. Different lowercase letters indicate significant differences.

**

**

**Figure S3.** Effect on the incidence of faba bean Fusarium wilt during different onset periods and intercropping effect. (A) 2016-2017 (planted for three years). (B) 2018-2019 (planted for five years). (C) 2020-2021 (planted for seven years). (D) 2021-2022 (planted for eight years). The values were the averages ± standard deviation by three biological replicates; Different lowercase letters indicated significant difference.





**Figure S4.** Effect on the disease index of faba bean Fusarium wilt during different onset periods and intercropping effect. (A) 2016-2017 (planted for three years). (B) 2018-2019 (planted for five years). (C) 2020-2021 (planted for seven years). (D) 2021-2022 (planted for eight years). The values were the averages ± standard deviation by three biological replicates; Different lowercase letters indicated significant difference.

As shown in Figure S3 and S4, under the M treatment in the same year, compared with the i treatment, the treatments of ii and iii significantly increased the incidence and disease index of faba bean Fusarium wilt. Under the i and ii treatments, with the increase of planted years, the incidence and incidence index of faba bean Fusarium wilt also increased.

Under the i and ii treatments in the same year, compared with the M treatment, the treatments of I significantly decreased the incidence of faba bean Fusarium wilt. Under the i, ii and iii treatments in the same year, compared with the M treatment, the treatments of I significantly decreased the disease index of faba bean Fusarium wilt. This analysis shows that the incidence and incidence index of faba bean increased with the onset time. However, the interaction between wheat and faba bean could significantly reduce the incidence of the disease and the incidence index of faba beans during the early stage and peak stage of onset. This was particularly true for the most significant regulation of faba beans during the early stages of the disease. The results showed that intercropping played an important role in the prevention of Fusarium wilt in practical production, which could prevent the occurrence of this wilt in advance and lay a solid foundation for comprehensive control during the later period.

**References**

Wu, H. S., Wang, Y., Zhang, C. Y., Gu, M., Liu, Y. X., Chen, G., Wang, H. J., Tang, Z., Mao, Z. S., and Shen, Q. R. (2009). Physiological and biochemical responses of in vitro *Fusarium oxysporum* f. sp. *niveum* to benzoic acid. Folia Microbiol. 54:115-122.
